# Preventing the Next Pandemic: The Case for Investing in Circulatory Health – A Global Coalition for Circulatory Health Position Paper

**DOI:** 10.5334/gh.1077

**Published:** 2021-10-12

**Authors:** The Global Coalition for Circulatory Health, Leslie Rae Ferat, Ryan Forrest, Kawaldip Sehmi, Raul D. Santos, David Stewart, Andrew J. M. Boulton, Beatriz Yáñez Jiménez, Phil Riley, Dylan Burger, Erika S. W. Jones, Maciej Tomaszewski, Maria Rita Milanese, Paul Laffin, Vivekanand Jha, Bettina Borisch, Michael Moore, Fausto J. Pinto, Daniel Piñeiro, Jean-Luc Eiselé, Daniel T. Lackland, Paul K. Whelton, Xin-Hua Zhang, Anna Stavdal, Donald Li, Richard Hobbs, Jeyaraj Durai Pandian, Michael Brainin, Valery Feigin

**Affiliations:** 1 Framework Convention Alliance for Tobacco Control, CA; 2 International Alliance of Patients’ Organizations, GB; 3 International Atherosclerosis Society, IT; 4 International Council of Nurses, CH; 5 International Diabetes Federation, BE; 6 International Society of Hypertension, GB; 7 International Society of Nephrology, BE; 8 World Federation of Public Health Associations, CH; 9 World Heart Federation, CH; 10 World Hypertension League, US; 11 World Hypertension League, CN; 12 World Organization of Family Doctors, BE; 13 World Stroke Organization, CH

**Keywords:** Health Emergency Preparedness, COVID-19, Noncommunicable Disease, NCD, Circulatory Health, Cardiovascular Disease, CVD, Stroke, Diabetes, Kidney Disease, Hypertension, Syndemic, Public Health, Policy

## Abstract

The Coronavirus Disease 2019 (COVID-19) has had a continuous and robust impact on world health. The resulting COVID-19 pandemic has had a devastating physical, mental and fiscal impact on the millions of people living with noncommunicable diseases (NCDs). In addition to older age, people living with CVD, stroke, obesity, diabetes, kidney disease, and hypertension are at a particularly greater risk for severe forms of COVID-19 and its consequences. Meta-analysis indicates that hypertension, diabetes, chronic kidney disease, and thrombotic complications have been observed as both the most prevalent and most dangerous co-morbidities in COVID-19 patients. And despite the nearly incalculable physical, mental, emotional, and economic toll of this pandemic, forthcoming public health figures continue to place cardiovascular disease as the number one cause of death across the globe in the year 2020. The world simply cannot wait for the next pandemic to invest in NCDs. Social determinants of health cannot be addressed only through the healthcare system, but a more holistic multisectoral approach with at its basis the Sustainable Development Goals (SDGs) is needed to truly address social and economic inequalities and build more resilient systems. Yet there is reason for hope: the 2019 UN Political Declaration on UHC provides a strong framework for building more resilient health systems, with explicit calls for investment in NCDs and references to fiscal policies that put such investment firmly within reach. By further cementing the importance of addressing circulatory health in a future Framework Convention on Emergency Preparedness, WHO Member States can take concrete steps towards a pandemic-free future. As the chief representatives of the global circulatory health community and patients, the Global Coalition for Circulatory Health calls for increased support for the healthcare workforce, global vaccine equity, embracing new models of care and digital health solutions, as well as fiscal policies on unhealthy commodities to support these investments.

## Introduction

### Context: why circulatory health, why now

The Coronavirus Disease 2019 (COVID-19) has had a continuous and robust impact on world health. The resulting COVID-19 pandemic has had a devastating physical, mental and fiscal impact on the millions of people living with noncommunicable diseases (NCDs), as they have a higher risk of severe illness and death from COVID-19. COVID-19 has been associated with an excess in all-cause and cardiovascular disease (CVD) mortality beyond that related to the infection itself and its immediate consequences [1,2]. Studies in the United Kingdom (UK) and United States of America (USA) have clearly shown increasing deaths from ischemic heart disease, stroke and hypertensive disease due to COVID-19 [1,2]. Overall, the impact has been greater in individuals with lower socioeconomic status, [2,3] even in high income nations [4].

In addition to older age, people living with CVD, stroke, obesity, diabetes, kidney disease, and hypertension are at a particularly greater risk for severe forms of COVID-19 and its consequences [5]. Simultaneously, the burden of COVID-19 and the measures necessary to retard its progression have had a significant impact upon health systems. Lockdowns, reduction in CVD-related visits to emergency units, as well as cancellation of medical appointments, laboratory tests and the consequent inadequate control of CVD risk factors have all been quoted as possible causes for indirect excess mortality due to SARS-CoV-2 infection [6]. Consequently, the lack of adequate control of CVD risk factors and rising rates of sedentary lifestyles, obesity and type 2 diabetes may herald an ominous long-term impact on CVD [6,7].

Recent data have shown a decline in the assessment of risk factors such as blood pressure and cholesterol, despite the increase in the use of novel and telemedicine resources [7]. There is particular concern regarding people with, or at a greater risk of, type 2 diabetes during the COVID-19 pandemic. People living with diabetes not only have a greater risk of severe disease and mortality from COVID-19, but it has also impacted the management of diabetes. Data from the UK indicate reduction in diagnosis due to disruption of the health care system, as well as inadequate monitoring of glucose and CVD risk factors in people with type 2 diabetes [8]. This may lead to an increased risk of CVD over the medium term. Similarly, people with advanced chronic kidney disease undergoing dialysis faced the dual challenge of being both at higher (up to 20 times greater than the general population) risk of infection from SARS-CoV-2 due to their inability to self-isolate because they require regular in-centre care surrounded by numerous other patients as well as staff and having a disproportionately higher level of suffering from adverse outcomes once infected [9].

Predictably, the impact of COVID-19 upon circulatory health will be of a greater extent and longer duration in middle and low-income countries due to late onset and expansion of vaccination [10]. In addition, these regions present a greater burden of CVD and risk factors than those with higher income [11], with previous deficiencies of health care systems and burgeoning intrinsic economic disparities may increase disease burden even more [12].

In conclusion, COVID-19 impacts health beyond complications of infectious diseases and the current and future impact upon circulatory health must be faced directly. It is of extreme importance to identify and adequately manage those at greater risk to mitigate the already elevated burden of circulatory disease, with the greatest impact felt in low- and middle-income world regions. As the chief representatives of the global circulatory health community and patients, the Global Coalition for Circulatory Health has a unique responsibility to draw policymakers’ attention to the tsunami of post-pandemic consequences lying in wait.

### Context: the importance of evidence-based science, recommendations, and education

COVID-19 has brought a massive stress test upon health care practice and science overall [13]. The severity of the disease, the initial uncertainty, absence of adequate evidence about its natural history, prevention, therapies, and unprecedented restrictions on modern social life together, contributed to the difficulties. Furthermore, the distribution of unfounded and unproven recommendations on social media fuelled by political agendas, conspiracy theorists as well as ill-informed or opportunistic doctors and scientists, has created a very difficult situation for health authorities, governments, and health care practitioners [14,15]. Specifically, inadequate evidence has led to use of ineffective and possibly harmful therapies and failure to use masks and maintain social distancing may have cost the lives of hundreds of thousands of people worldwide [16,17].

Fortunately, randomized clinical trials have clearly shown what works or not in COVID-19 and vaccines are changing the face of the pandemic. On the positive side, the scientific community and industry has demonstrated our capacity to develop and bring to the market, in a few weeks or months, detection kits using different technologies (PCR, antigenic) and, in record time, effective vaccines, the most innovative ones based on mRNA technologies. Even if the access and distribution of billions of new products simultaneously worldwide is still a burning issue, industry has demonstrated in this particular case our capacity to fight effectively and very rapidly a new virus. However, lessons must be learned for the future on how authorities must deal with such enormous challenges, including the availability and affordability of vaccines and essential therapies.

COVID-19 has impacted health in several different ways and those at risk of or already living with CVD are at an especially heightened risk. Indeed, the excess mortality risk due to COVID-19 was comprised not only of consequences of infectious diseases but also those related to the cardiovascular system [1,2]. Fortunately, where the latter is concerned, there is robust evidence from randomized controlled studies that control of risk factors like dyslipidaemias, hypertension, smoking and diabetes can reduce the burden of circulatory diseases [18]. Though the Global Coalition for Circulatory Health acknowledges that emerging science does not always indicate a single course of action and political decisions will necessitate certain trade-offs, the Coalition fully endorses the use of adequate science and robust evidence-based medicine to guide its recommendations and educational programs to mitigate the burden of CVD in the post-COVID-19 era.

## The COVID-19 pandemic and circulatory disease

### Interruptions to services, access to care

During the evolution of the pandemic, risk factors for hospitalisation, severe complications of acute infection, ICU admission and death have been observed. The OpenSAFELY platform documented, across a large database of adults in the UK that chronic cardiac disease, stroke, dementia, reduced kidney function, uncontrolled diabetes and organ transplant considerably increase the risk of death in patients with a positive diagnosis of COVID 19 [19]. While OpenSAFELY shows that hypertension itself does not necessarily increase the risk for severe disease and death from COVID-19, many CVD patients also live with multiple coexisting comorbidities, which make them even more vulnerable to COVID-19 [20], and hypertension was observed as the most frequent comorbidity in patients who died from COVID-19 [21]. Diabetes with uncontrolled hyperglycaemia significantly increases the risk of severe COVID-19 as well as mortality, compared to the cohorts without diabetes, hyperglycaemia, or obesity [22]. Such weighted risk factors have also been used to produce QCOVID, a validated digital risk score, for use by practitioners/health systems analogous to CVD prevention scores [23].

International lockdown protocols, access to regular coronavirus reports and updates and overwhelmed healthcare services have resulted in a decline in individuals accessing healthcare services for non-COVID related conditions [4,5,6,24,25,26]. The impact on the diagnosis, management and ongoing treatment of chronic conditions has left many people extremely vulnerable to complications. In many Low- and Middle-Income Countries (LMICs), the provision of in-centre dialysis was severely reduced during lockdowns resulting in patients needing dialysis not being able to receive their treatment [27,28]. While there has been a decline in hospital visits for acute myocardial infarctions [25,29] heart failure and in-centre dialysis there has been a rise in out-of-hospital deaths [27,28,30].

A survey of 1050 patients in the UK with heart failure found that 32% were reluctant to access healthcare systems and 65% reported that appointments were cancelled or postponed during lockdown [31]. A report from Uganda has demonstrated the inequalities in healthcare between HIV and hypertension. In people with a combination of HIV and hypertension 92–100% could access their antiretrovirals in alternative health facilities, whereas only 4–8% could access antihypertensive medication as well [32]. People with chronic conditions (hypertension, stroke, diabetes, kidney disease and heart disease) had difficulty in accessing healthcare while lockdown led to sedentary lifestyle with increased stress and anxiety in a study from India [33]. A study from the US observed an apparent increase in deaths due to diabetes during the pandemic, which suggests an indirect impact of COVID-19 on routine diabetes care (hesitation in seeking medical attention in hospitals, patients discharged prematurely due to overwhelmed healthcare facilities, restrictions in outpatient care for diabetes, potential delays in emergency care) [34].

Inequalities in health care were brought to the fore by the COVID-19 pandemic. The decline in non-COVID related hospital admissions was greater in areas of resource constraints as was the decline in blood pressure control in people of colour [35]. Furthermore, there is less information available from LMICs with only a third of the publications in a recent review stemming from LMICs [25]. Access to telehealth or remote healthcare (e.g., home blood pressure monitoring and telemedicine consultations on glycaemic control for diabetes patients) is not necessarily able to alleviate these disparities because it often comes with the expense of extra personal equipment.

### Multimorbidities and the Global NCD agenda

The combined impacts of cardiovascular complications due to COVID-19 and interruptions to crucial medical interventions and ongoing care for people living with hypertension, diabetes, kidney disease, stroke, and other circulatory conditions – those most at risk of poor outcomes from COVID-19 – will exacerbate the already huge burden borne by stressed health systems worldwide.

To better understand the extent of disruptions to essential health services caused by the COVID-19 pandemic, in early 2021 the World Health Organization (WHO) launched the second round of the *National pulse survey on continuity of essential health services during the COVID-19 pandemic*. The survey presents global findings from the 135 countries and territories that participated in the second round of the survey during January-March 2021. The findings offer critical insight from country key informants into the extent of impact of the COVID-19 pandemic on essential health services across the life course, the reasons for those disruptions, and how countries are adapting strategies and approaches to maintain service delivery.

This survey follows up on the WHO’s pulse surveys distributed in 2020, including: Pulse survey on continuity of essential health services during the COVID-19 pandemic; Rapid assessment on the impact of the COVID-19 pandemic on noncommunicable disease resources and services; Rapid assessment on the impact of COVID-19 on mental, neurological and substance use services; and pulse surveys on immunization (Round 1 and Round 2).

The surveys together found that while nearly every participating Member State reported moderate to severe disruptions to essential services for NCDs, only 38% had explicitly included NCDs in their response and preparedness plans as prescribed by the 2020 World Health Assembly Resolution, while only 3% had explicitly allocated funds for these efforts.

It is therefore essential that governments and medical federations work across disease silos to speak with one voice for better integration of NCD services in emergency preparedness plans. Together, the organizations comprising the Global Coalition for Circulatory Health can reach tens or even hundreds of thousands of physicians, advocates, and members of the healthcare workforce around the world; all of whom have a part to play in the reduction of the compounding negative impacts of insufficient emergency preparedness on both circulatory and broader medical health. The convergence of other sectors and movements with a relevant role – from research and development to the manufacture of Protective Personal Equipment – contributes to the unique opportunity for action presented in the post-COVID era.

## Emergency response

### Supporting the health workforce

A number of crucial issues must be addressed if WHO Member States are to create more robust and resilient health workforces. To begin, most of the global health workforce are women working on the front line. The combination of direct exposure to the virus, violence, and stigma, in addition to the double burden of care, has made it clear that gender must be taken into account when we plan how to support and protect the workforce in future health crises.

Accessibility to Personal Protection Equipment (PPE) should be considered a minimum requirement, but—just as important—we must plan service delivery in teams and develop health systems which protect those who are exposed to the heaviest burden, both in primary and in secondary care.

Member states also need to address the imbalance of the workforce. There is an increasing amount of evidence indicating that primary care, manned with multidisciplinary teams, is a requirement for resilient health systems. The COVID-19 pandemic has made it even more evident and pressing that resilient health systems require a strong primary care with multidisciplinary teams. High priority should be placed on addressing the imbalance of the workforce to ensure provision of efficient and high-quality primary care to all people.

For effective prevention, screening and triage, measures should be tailored to local contexts and address the needs related to an ageing population and the NCD epidemic, which was on the rise long before the current pandemic. Triage, screening, and gatekeeping are necessary to provide people with the right level of care and to avoid unnecessary hospital admissions. As secondary care continues to dominate in medical and nursing schools globally, it is crucial that undergraduate and postgraduate training be reformed and take place in primary care settings. Medical education should also be strengthened with the teaching of ‘cultural humility,’ which is the ‘ability to maintain an interpersonal stance that is other-oriented (or open to the other) concerning aspects of cultural identity that are most important to the [person]’ [36], so to better prepare health care workers to provide indigenous populations with appropriate and effective treatments, as they often face several barriers in accessing care, including racism within the health care system, stigma, and ethnic bias, while having a higher prevalence of CVD compared to the general population.

Policymakers need to change focus from institutional, acute care to community based, integrated, and personalized care. To attain this, support from hospitals and specialized care is paramount to retain healthcare workers in the communities.

### Vaccine equity and People Living with NCDs (PLWNCDs)

Living with COVID-19 has disrupted healthcare systems, leading to delays in healthcare provisions, a decrease in referrals for secondary care, disruptions of organ transplantation services and live donations. Emergency planning should take these key needs into account, whether the cause in disruption is a natural or human caused disaster, or a pandemic such as COVID.

The breakdown of health systems and disruptions due to lockdowns have had a severe impact on the ability of people living with diabetes, hypertension, kidney disease, stroke and other circulatory conditions and people with malignancies to access regular care, putting them at greater risk of poor health outcomes from COVID-19, and further exacerbating the already huge burden borne by health systems worldwide. For patients undergoing dialysis, even a brief interruption in chronic dialysis treatment is a death sentence, and patients with a kidney transplant may experience transplant rejection if deprived of immunosuppressive medications. Furthermore, COVID-19 has disrupted healthcare systems, leading to delays in healthcare provisions, a decrease in referrals for secondary care, disruptions of organ transplantation services and live donations.

Now that vaccines against COVID-19 are available, distribution and access to the vaccines should take very high priority. Sadly, COVID-19 has also exacerbated the shocking inequalities between High Income Countries (HIC) and LMICs, where four out of five people with an NCD live, including for the provision of vaccines. While the majority of HICs, having access to enough doses to vaccinate their populations, have put in place advanced and extensive vaccination programs, most LMICs are still being left behind. This inequity in vaccine distribution is leaving millions of people vulnerable to the virus and allowing new variants to emerge and spread across the world, leading countries with advanced vaccination rates to reinforce new public health measures and restrictions. In turn, the COVID-19 pandemic is further widening economic disparities between countries, which will bring negative repercussions for all. As remarked by the UN Secretary-General António Guterres while speaking at the European Parliament in Brussels ‘Vaccines are our only way out of this crisis. They must be considered as a global public good, available and affordable to all,’ adding that ‘Vaccine equity is not only the greatest moral test of our times. It is also a matter of effectiveness.’

Ensuring equitable access to vaccines is of vital importance to end the pandemic and prevent millions of deaths. This requires extraordinary measures and global collaboration. International support and adequate funding to programs aimed at collaborating to accelerate development, production, and equitable access to COVID-19 tests, treatments, and vaccines, such as the Access to COVID-19 Tools (ACT) Accelerator, are critical, alongside sharing technology and manufacturing know-how, to ensuring equitable access to vaccines.

Following a call from the G20 leaders in March 2020, the ACT Accelerator was launched in April 2020 by the WHO, European Commission, France and The Bill & Melinda Gates Foundation. It brings together governments, scientists, businesses, civil society, and global health organizations, focusing on four pillars for equitable distribution of COVID-19 tools to those countries most in need: Diagnostics, Therapeutics and Vaccines (also known as COVAX), with the Health Systems Connector pillar working across the other three [37]. COVAX, the vaccines pillar of the ACT Accelerator, aims to ensure that every country receives fair and equitable access to safe and effective COVID-19 vaccines.

As people living with NCDs are at higher risk of negative health outcomes from COVID-19 due to their impaired immune systems and presence of co-morbidities, particularly people on dialysis and transplantation, as these are the leading global risk factors for death from COVID-19 [38], global efforts need to be put in place to deploy vaccination efficiently and equitably to these vulnerable and high-risk groups in all countries [9]. In addition, it is recommended that early vaccination should be prioritized for these populations, simultaneously with the elderly, and ‘administered regardless of whether patients have previously had COVID-19 or have positive IgG titres for SARS-CoV-2’ [39].

While the development of COVID-19 vaccines has been extraordinarily fast, the current supply cannot match the demand. Therefore, the global effort to respond to COVID-19 needs not only to include innovations in international supply chain to distribute vaccines globally, but also to boost LMICs’ capacity to produce the vaccines locally.

The Global Coalition for Circulatory Health strongly endorses the 74^th^ World Health Assembly resolution on Strengthening local production of medicines and other health technologies to improve access [40] and its call to Member States to ‘strengthen their leadership, commitment and support in promoting the establishment and strengthening of quality and sustainable local production of medicines and other health technologies,’ ‘further engaging in North–South and South–South development cooperation, partnerships and networks to build and improve the transfer of technology related to health innovation.’ Building capacity for local production of vaccines in LMICs, including voluntary transfer of intellectual property and know-how, through initiatives such as the WHO COVID-19 Technology Access Pool (C-TAP), is essential to scale up vaccination efforts.

Global equitable access to vaccines is thus an urgent and critical need not only to protect vulnerable people living with NCDs and health care workers, but also to mitigate the public health and economic impact of the pandemic, as no one is safe until everyone is safe.

## Emergency preparedness

### Transitioning to new models of quality circulatory health care: telemedicine

The challenges presented by the COVID-19 pandemic on circulatory health care are distinctive and constantly evolving. Such challenges include a shift towards sedentary behaviours, reduced access to health care providers, resource restrictions, and delayed or non-treatment. This has, in turn, stimulated flexibility and innovation in models of CV health care delivery to address patient needs; these innovations will, in all likelihood, extend beyond the COVID-19 pandemic and the specific locales in which they have been used. Such a transition offers an unprecedented opportunity to bridge historical care gaps and improve global circulatory health care.

In particular, there has been an increase in the use of telemedicine (via telephone or video) for circulatory health care delivery [41]. Telemedicine offers potential to address longstanding inequities in access to global circulatory health care. In the short term, telemedicine maintains links between health care providers and patients, while complying with social distancing and self-isolation requirements. Extending beyond pandemic times, telemedicine is likely to improve or initiate care delivery for individuals with mobility issues and those in remote or underserved communities.

Given the potential for expanded health care and the associated cost savings [41], we are likely to see incorporation of telemedicine into modern circulatory care going forward. It is therefore critical in this transition to ensure equitable access and care delivery still adheres to best clinical practices. Presently, data in this area are limited, however a recent American study showed that female, non–English-speaking, older, and poorer patients were less likely to access remote care options suggesting possible issues in equitable access [42]. Such issues are more pronounced in low-income countries (LICs), and LMICs [43]. A second study corroborated several of these findings but also showed that providers were less likely to prescribe medicines or order diagnostic tests during telemedicine visits compared to in-person visits [44]. While the reasons for these observations are unclear, they suggest that remote cardiovascular care can contribute to or maintain care gaps and continued optimization of telemedicine delivery is needed to deliver its promise.

### Transition to new models of quality circulatory health care: self-monitoring

High blood pressure is the most important reversible risk factor for recurrent stroke, with relative risk increasing by about one third for every 10mmHg increase in systolic blood pressure [45]. People who have survived previous stroke or transient ischaemic attack (TIA) are at particularly high risk of subsequent stroke [46]. One of the key reasons for this is that control of blood pressure is frequently sub-optimal with significant proportions of individuals remaining above target levels recommended in guidelines [47,48,49]. Potentially modifiable reasons for poor control include clinical inertia, poor adherence to medication, organizational failure, cost of healthcare services and medicines, and lack of engagement of carers. Carers can play a key role in supporting adherence after stroke [50], but previous trials of interventions to improve adherence with antihypertensives post stroke have failed to consider this [51]. Solutions need to address all four factors [52,53,54,55].

Most management of hypertension is undertaken in primary care, and it is therefore necessary for interventions to be delivered in this setting [56]. Given the increasing workload demands on primary care, interventions need to ideally reduce, not increase, workload [57]. Self-management is potentially attractive in this regard but not all patients want/are able to do this.

It has been demonstrated that General Practitioner (GP) supervised self-monitoring and self-management solutions are effective at lowering blood pressure in primary care [58,59,60]. These appear to work by improving patient adherence and increasing appropriate prescription of antihypertensive medication (reduction of clinical inertia) [51,61]. Multiple BP measures at home provide better estimates of long-term risk than clinic readings [62]. Technological advances mean that even basic mobile phones can be used to transmit results to supervising clinicians with simple reports incorporated into routine practice data therefore potentially revolutionising the organization of care [63]. Such phones are not commonplace for all age groups or across all resource settings: as of 2015, 93% own and use a mobile phone, 83% of those 65–74, 50% of those over 75 [64]. Smart phone use is fast increasing with 70% of adults now using one, albeit with lower market penetrance in older people (2013: 20% 65–75, 5% 75+; 2015: 28% 65–74, 8% >75). Similar numbers of those over 65 use tablets or laptops to access the internet as opposed to desktop computers. Monitoring clinicians can contact users as necessary, and users can be automatically informed by text, app notification or email of the rolling average of their results, their level of control and advised if they need to adjust their medication or contact their clinician.

Given the potential mobility problems that can follow stroke, interventions reducing the need to travel to GP surgeries are appropriate for this population. Increasing GP workload also means that reducing the need for home visits and potentially improving efficiency via greater use of tele-monitoring systems has face validity.

Self-monitoring/management is not a universal panacea however and appears to have reduced impact in resistant hypertension: our individual patient data meta-analysis of self-monitoring suggests reduced effectiveness in those with very high baseline blood pressure and/or multiple medications. Reduced adherence to medication is the major cause of resistant hypertension and can be detected by simple urine assays [65]. Identification of such issues earlier in the care pathway might significantly improve control and reduce workload by facilitating discussion and active management of barriers. The evidence for patient self-management being cost effective (57, 59) is particularly important for countries with dispersed and rural populations, but also wherever access to healthcare is limited or difficult or becomes disrupted (as during a pandemic).

### Transitioning to new models of quality circulatory health care: patient co-creation

As public health has developed as a discipline, patients and patient representative groups have consistently advocated that nothing should be decided or produced without their engagement in the decision-making and creation process: nothing about us without us.’ Patient engagement must be employed along the entire value chain of healthcare and across the full spectrum of healthcare services, including promotion, prevention, treatment, rehabilitation, and palliation.

Over the last decade a group of uber patient advocates have been working with enlightened health systems and pharmaceutical companies to support the integration of healthcare value chains by engaging at the very start of the value chain in the research and development of medicines, health devices and services. This engagement then matures and progresses onwards into collaborating with regulators and even the health technology assessment bodies to ensure patients have timely access to safe, quality and patient centric innovative medicines, vaccines, gene and cell therapies and medical devices.

Co-production taps into perspectives and insights of patients and carers with ‘lived experiences’ of one or more particular conditions. They are often best placed to comment and advise on what medicines, support and services make a positive difference to their lives. If it is all done well, co-production helps to ground discussions, and to maintain a patient-centred perspective. Co-production, when extended into the ecosystem surrounding the pandemic, must be a part of a range of approaches that should include citizen involvement, participation, engagement, and consultation. It can become the cornerstone of self-care, of patient-centred care approaches in future pandemics.

Public health systems, especially during an emergency, have traditionally always adopted a top-down and centrally controlled response. During the early stages of the pandemic this central response tended to ignore patient engagement and many countries used their public health’s legal, policy and practice to institute and enforce lockdowns that adversely impact patients as their access to treatment and support was disrupted. Trending as #LockdownsWithOutPlan on the social media, it was clear from the postings that most shielding programmes were creating severe hardship and morbidity among patient groups.

At the 74^th^ World Health Assembly, the European Union and 29 other Member States proposed the Resolution WHA 17.3 Strengthening WHO preparedness for and response to health emergencies. The resolution has called for a whole-of-government and whole-of-society response within the Member States and proposed that there should be a permanent mechanism and framework set for the coordination and inclusive collaboration among all stakeholders during public health emergencies.

The World Health Assembly has accepted that they will set up a Member States’ Working Group on Strengthening WHO preparedness and response to health emergencies and this will be open to all Member States as well as work with other relevant bodies, organizations, non-State actors and any others with relevant information and experience. Patient groups have an open invite and clear mandate to participate in this body. The WHO has also demonstrated its commitment to progress through initiatives like the inauguration of its new Hub for Pandemic and Epidemic Intelligence in Berlin. It is essential that civil society organizations, patient representative groups, United Nations agencies, and Member States work together to collaboratively build back better and coproduce a robust pandemic prepared global health governance and control system through this framework if they are to prevent a similar syndemic in the future.

### Fiscal policies for health

Tobacco use, alcohol use, and consumption of unhealthy foods (such as sugar-sweetened beverages [SSBs], or food artificially high in salt) are leading risk factors for the development of NCDs, including hypertension, diabetes, kidney disease, stroke, and other circulatory conditions. These are the very same underlying conditions that have put so many people at an increased risk of severe illness and death from COVID-19.

The co-occurring and interlinked nature of CVD and COVID-19 pandemics has made it clear that recovery from COVID-19 and future preparedness will require concerted action to address underlying risk factors for CVD, including through greater investment in disease prevention and health promotion policies. To date, these measures have largely been left out of conversations about pandemic preparedness [66]. Fiscal policies, in particular – including taxes on health-harming commodities like tobacco, alcohol, and sugar-sweetened beverages (SSBs) – have a critical role to play in ‘building back better’ a supporting future pandemic preparedness.

There is strong global evidence to indicate that excise taxes are highly effective on three levels: first, at reducing the consumption of tobacco, alcohol, and SSBs in both high- and low-income countries, second, saving on healthcare expenditure, and third, simultaneously boosting government revenues [67]. Estimates suggest that a global increase in excise taxes to raise the prices on alcohol, tobacco, and SSBs by 20% over 50 years could avert more than 18 million premature deaths, while at the same time increasing government revenues by US$1987billion [68]. The health benefits of increased excise taxes on these unhealthy products would go a long way towards investments in Universal Health Coverage (UHC), supporting future pandemic preparedness (including by reducing the NCD-related burden on health care systems), and creating healthier populations which are more resilient to future infectious disease outbreaks or pandemics. In addition, this would respond to the very aggressive marketing strategies of unhealthy commodity industries, such as linking their products with the work of health professionals, emergency services, and other frontline workers during the pandemic.

The revenue-generation potential of health taxes is also of critical importance during this time. Governments, saddled with large budget deficits as a result of last year’s economic downturn [69], must find the fiscal space for continued public spending on essential health services and social supports as well as investment in future pandemic preparedness. Raising health taxes could help to cover the costs of this spending.

A recent study by the Centre for Global Development estimates that increasing taxes on tobacco, alcohol, and SSBs could halve revenue shortfalls associated with increased spending stemming from the pandemic in LMICs [70]. Policymakers might also consider earmarking revenues from health taxes for spending on health promotion and preventive measures. Already a small number of countries have begun to explore this practice as a way to prioritize resources for health during the COVID-19 pandemic [71].

## Summary of recommendations

The COVID-19 pandemic has strong negative influence on circulatory diseases, especially for patients with vascular risk factors and NCDs, and this includes access to all medical care facilities such as primary care, acute services or after care and rehabilitation. The Global Coalition for Circulatory Health is a global network of professional federations and organizations that works to speak with one voice for better NCD services, including emergency preparedness for all patients with vascular diseases including CVD, stroke, hypertensive diseases, and others. The Coalition has noted shocking inequalities in availability of vaccines and essential medicines to fight this pandemic between HICs and LMICs, the situation in the latter being most dramatic, and strongly endorses the 74th WHA Resolution on strengthening local production of medicines and other health technologies.

Furthermore, the Global Coalition for Circulatory Health recommends the following:

- As a first step, prevent, screen, and treat for circulatory conditions through national COVID-19 response and recovery plans via concerted patient co-creation and collaboration.
- Increase spending and develop targeted policies to tackle CVD and NCD risk factors, including the social and commercial determinants of health, using revenues from fiscal policies (i.e., taxation of unhealthy commodities, such alcohol and tobacco products).
- Include indicators on circulatory disease prevalence, comorbidities, and risk factors into measures of pandemic readiness, resilience, and response.
- Ensure people living with circulatory conditions and in low-resource settings have good and equitable access to essential health services, including medicines, supplies and associated devices, through Primary Health Care.
- Provide easy priority access to vaccination and other disease prevention methods for those with underlying circulatory risk factors.
- Support and integrate the use of effective new models to deliver quality health services, especially telemedicine and initiatives to support self-care and self-empowerment.

## Conclusion: the case for investing in NCD screening/services/integration

In his September 2020 editorial in *The Lancet*, Editor-in-Chief Richard Horton boldly asserted that the global medical community was not in the midst of fighting a *pan*demic, but rather a *syn*demic – the devastating aggregate consequence of biological and societal interactions that impact health processes and outcomes. The relationship between NCDs and COVID-19 is multifaceted and complex, but it has become clear that ‘In the case of COVID-19, attacking NCDs will be a prerequisite for successful containment [72].’

This is particularly true for circulatory health conditions. Meta-analysis indicates that hypertension, diabetes, chronic kidney disease, and thrombotic complications have been observed as both the most prevalent and most dangerous co-morbidities in COVID-19 patients [73]. And despite the nearly incalculable physical, mental, emotional, and economic toll of this pandemic, forthcoming public health figures continue to place cardiovascular disease as the number one cause of death across the globe in the year 2020 [74].

The world simply cannot wait for the next pandemic to invest in NCDs. Social determinants of health cannot be addressed only through the healthcare system, but a more holistic multisectoral approach with at its basis the Sustainable Development Goals (SDGs) is needed to truly address social and economic inequalities and build more resilient systems. Yet there is reason for hope: the 2019 UN Political Declaration on UHC provides a strong framework for building more resilient health systems, with explicit calls for investment in NCDs and references to fiscal policies that put such investment firmly within reach. By further cementing the importance of addressing circulatory health in a future Framework Convention on Emergency Preparedness, WHO Member States can take concrete steps towards a pandemic-free future.
